# Prioritisation of patients awaiting hip and knee arthroplasty: Lower pre‐operative EQ‐5D is associated with greater improvement in quality of life and joint function

**DOI:** 10.1002/msc.1645

**Published:** 2022-05-13

**Authors:** Luke Farrow, James Redmore, Partha Talukdar, Nick Clement, George P. Ashcroft

**Affiliations:** ^1^ Institute of Applied Health Sciences University of Aberdeen Aberdeen UK; ^2^ Grampian Orthopaedics Woodend Hospital Aberdeen UK; ^3^ Trauma & Orthopaedics Royal Infirmary of Edinburgh Edinburgh UK; ^4^ Trauma & Orthopaedics Edinburgh Orthopaedics Edinburgh UK

**Keywords:** arthritics, quality of life

## Abstract

**Introduction:**

The COVID‐19 pandemic has led to unprecedented delays for those awaiting elective hip and knee arthroplasty. Current demand far exceeds available resource, and therefore it is integral that healthcare resource is fairly rationed to those who need it most. We therefore set out to determine if pre‐operative health‐related quality of life assessment (HRQoL) could be used to triage arthroplasty waiting lists.

**Methods:**

Data regarding demographics, perioperative variables and patient reported outcome measures (PROMs) (pre‐operative and 1‐year post‐operative EuroQOL five dimension (EQ‐5D‐3L) and Oxford hip and knee scores (OHS/OKS) were retrospectively extracted from electronic patient health records at a large university teaching hospital. Patients were split into two equal groups based on pre‐operative EQ‐5D TTO scores and compared (Group1 [worse HRQoL] = −0.239 to 0.487; Group2 [better HRQoL] = 0.516–1 [best]).

**Results:**

513 patients were included. Patients in Group1 had significantly greater improvement in post‐operative EQ‐5D‐3L scores compared to Group2 (Median 0.67 vs. 0.19; *p* < 0.0001), as well as greater improvement in OHS/OKS (Mean 22.4 vs. 16.4; *p* < 0.0001). Those in Group2 were significantly less likely to achieve the EQ‐5D‐3L minimum clinically important difference (MCID) attainment (OR 0.13, 95%CI 0.07–0.23; *p* < 0.0001) with a trend towards lower OHS/OKS MCID attainment (OR 0.66, 95%CI 0.37–1.19; *p* = 0.168). There was no clinically significant difference in length of stay (Median 3‐days both groups), and no statistically significant difference in adverse events (30 days and 1 year readmission/reoperation).

**Conclusions:**

A pre‐operative EQ‐5D‐3L cut‐off of ≤0.487 for hip and knee arthroplasty prioritisation may help to maximise clinical utility and cost‐effectiveness in a limited resource setting post COVID‐19.

## 1 INTRODUCTION

The coronavirus (COVID‐19) pandemic has had a significant detrimental impact on access to elective healthcare services, in particular orthopaedic surgical procedures such as hip and knee arthroplasty (COVIDSurg Collaborative, 2020; Farrow et al., 2021). There is now a historical backlog of patients awaiting elective orthopaedic surgery (Carr et al., 2021; Oussedik et al., 2021), many of whom are suffering with progression of their symptoms because of delays to life changing operations (Clement et al., 2021; Learmonth et al., 2007).

Accordingly waiting lists have risen drastically, as demand far outstrips available capacity. For example, the number of patients waiting more than 1 year for elective surgery in England rose from 1000 in 2019 to just under 140,000 at the beginning of 2021 (Carr et al., 2021). Even with a sharp increase in productivity compared to pre‐pandemic levels the deficit will take several years to clear (Yapp et al., 2021). Furthermore, current evidence suggests that elective activity remains far short of parity with previous throughput (NHS England, 2021 https://www.england.nhs.uk/wp‐content/uploads/2021/03/B0468‐implementation‐guidance‐21‐22‐priorities‐and‐operational‐planning‐guidance.pdf).

Historically the primary method of prioritising patients on the routine waiting list for hip and knee replacement was through time waited (Gutacker et al., 2016). Alternatively, patients could be prioritised according to their health‐related quality of life (HRQoL), with those enduring a worse health state being prioritised.

Previous research has highlighted a distinct bimodal distribution of pre‐operative HRQoL in patients awaiting hip and knee arthroplasty as measured through the EuroQOL five dimension (EQ‐5D‐3L) time trade‐off (TTO) score (Clement et al., 2021). Its potential use to help prioritise patients for hip and knee arthroplasty surgery is however currently unexplored.

The primary aim of this study was to determine if patients with a worse HRQoL had a greater chance of attaining a clinically significant improvement in their HRQoL, compared to those with a better HRQoL. Secondary aims were to assess following primary total hip arthroplasty (THA) or total knee arthroplasty (TKA) compared to in terms of their demographic characteristics and clinical outcomes according to level of HRQoL preoperatively.

## 2 METHODS

### 2.1 Study design, setting and participants

A retrospective cohort study was performed utilising data from a single large University teaching hospital within Scotland. Information regarding patients undergoing elective primary hip and knee arthroplasty procedures (THA; TKA; Unicompartmental knee arthroplasty [UKA]) were obtained from electronic health records. This included patients from Q1 of 2017 to Q1 2019, in order to allow for obtainment of 1‐year HRQoL data at the time of data capture (March 2021) without any indirect influence from the COVID‐19 pandemic. Only patients with complete data regarding pre‐ and post‐operative HRQoL (EQ‐5D‐3L and Oxford hip and knee scores [OHS/OKS]) were eligible for inclusion. Exclusion criteria included those undergoing Revision Hip and Knee Arthroplasty, Emergency surgery (e.g., due to hip fracture) or Arthroplasty surgery at other anatomical sites.

### 2.2 Data collection

Data was obtained from two main sources: the patient electronic health record (demographic information and outcomes) and a local patient reported outcome measures (PROMs) database, linked via a separately held secure key identifier and pseudonymised. A preformatted data collection proforma was utilised to ensure appropriate capture of all key variables. These included:

Demographics—age, gender, past medical history of anxiety and/or depression, employment status (employed/unemployed/retired), ASA grade, BMI, Scottish index of multiple deprivation (SIMD) decile.

Outcomes—length of stay, hospital readmission (30 days and 1 year post‐operatively), reoperation (30 days and 1 year post‐operatively), and venous thromboembolism (VTE) (30 days and 1 year post‐operatively).

Generalised and joint specific HRQoL PROMs EQ‐5D‐3L (EuroQol Group, 1990) and OHS/OKS (Murray et al., 2007; pre‐operative and 1 year post‐operatively). These are routinely collected as part of a PROMs programme within the NHS.

The primary outcome for the study was attainment of the minimum clinically important difference (MCID) in the EQ‐5D‐3L TTO score at 1‐year post‐arthroplasty. The EQ‐5D‐3L TTO is a widely used and validated HRQoL measure that generates single values for different combinations of health‐states based upon how individuals compare *x* years of healthy living to *x* years of illness. The MCID for EQ‐5D‐3L TTO has been previously evaluated in the literature and determined to be a ≥0.08 improvement post‐operatively (Luo et al., 2010).

Secondary outcomes included pre‐ and post‐operative changes in TTO valuation scores and OHS/OKS scores, OHS/OKS MCID attainment (set at >5 for both OHS and OKS according to prior literature [Clement et al., 2014; Yeo et al., 2020]), length of stay and clinical outcomes (readmission and reoperation).

### 2.3 Sample size calculation

An *a priori* sample size calculation was performed using a freely available online tool (https://clincalc.com/stats/samplesize.aspx). This calculation indicated a minimum of 398 total patients (199 per group) to detect a 10% difference in EQ‐5D‐3L TTO MCID attainment (clinically significant improvement in HRQoL) between two groups at 80% power and *p* < 0.05.

There were 1198 potentially eligible patients for inclusion from the linked data sources. The study cohort was identified using a random subset of minimum 500 patients selected using an online random sequence generator and stratified to include similar proportions of hip and knee patients.

### 2.4 Data processing and statistical analysis

Initial data scoping identified minimal missing data, with no evidence of missing data not at random and therefore no formal data imputation techniques were utilised. Pairwise deletion was performed to manage missing data fields, with no patient excluded due to missing data.

EQ‐5D‐3L TTO scores were generated from a freely available excel calculator utilising the pre‐determined valuation scores specific to the UK population (Dolan, 1997), as a composite score of overall HRQoL including the five domains assessed by the EQ‐5D (Activity level, anxiety, mobility, pain and self‐care).

**FIGURE 1:**
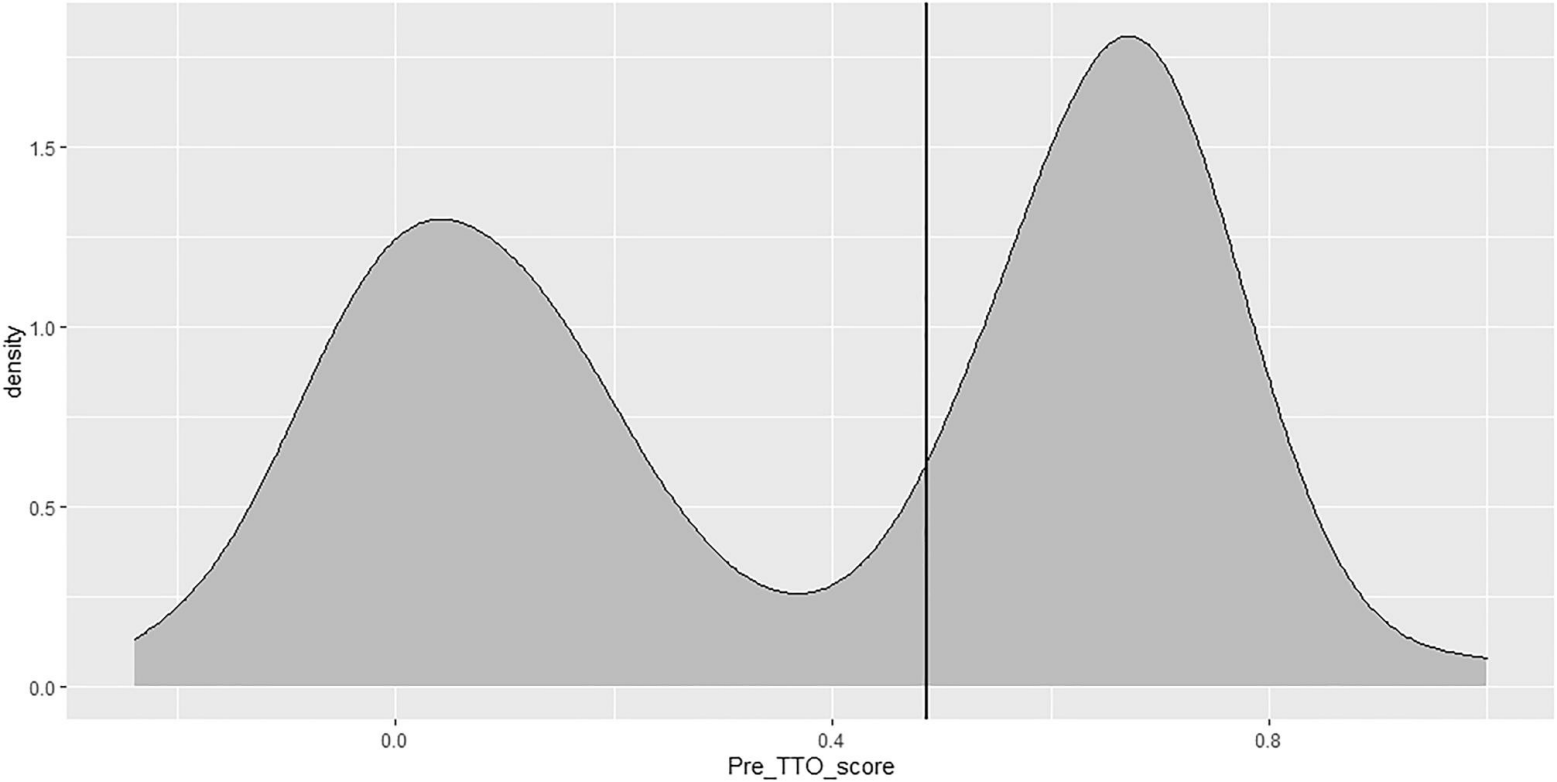
Density plot for pre‐operative EQ‐5D values

Following generation of the EQ‐5D‐3L TTO scores we dichotomised the study population into two groups based on their TTO scores, in line with previous work highlighting a binomial distribution in scores. We utilised the median value of TTO score to determine the cut off value, which was 0.516. This formed the lower limit for those with a better HRQoL (Group 2), with the higher limit set at 1, the maximum possible EQ‐5D‐3L TTO score. Given the non‐continuous nature of EQ‐5D score distributions the upper limit for those with a worse HRQoL (Group 1) was set at 0.487 and the lower limit −0.594 (the lowest possible EQ‐5D‐3L TTO score), though the lowest score seen in this study was −0.239. An illustration of the binominal distribution of the EQ‐5D‐3L TTO scores within the included study population is shown in Figure 1.

Assessment included baseline comparison of covariates between the two groups, as well as comparative analysis of both clinical and HRQoL measures as determined in the primary and secondary outcomes. Student *t*‐tests were used for normally distributed continuous data, Mann–Whitney U tests for non‐normally distributed continuous data, logistic regression for binomial data and chi squared testing for categorical data with multiple groups (including Bonferroni adjustment).

For the primary outcome an additional multivariable logistic regression was performed to identify the potential impact of pre‐identified clinically relevant potential confounders (age, gender, history of anxiety/depression, work status, ASA grade, SIMD and pre‐operative OHS/OKS scores).

All statistical analyses were performed using SPSS for Windows (version 24.0, SPSS Inc.). In all analyses *p* < 0.05 denoted statistical significance. Figures presented were created using the package ggplot in R statistics.

### 2.5 Ethics

Due to the retrospective, pseudonymised nature of data collection at our single institution, formal ethical approval was not required. Our study was however conducted in accordance with the 1964 Helsinki declaration and its later amendments. The project was reviewed and approved by our local PROMs data governance team, with trust registration as a service evaluation project performed. Data storage and analysis was undertaken in alignment with the Caldicott principles. There was no direct external funding source for the study. The study has been reported according to the strengthening the reporting of observational studies in epidemiology (STROBE) statement.

## 3 RESULTS

### 3.1 Demographics

A total of 513 patients (57.7% female) were included, with 244 in Group 1 (lower pre‐operative HRQoL) and 269 in Group 2 (higher pre‐operative HRQoL). The mean age of included individuals was 69.4 (interquartile range [IQR] 63–77). Overall, the two groups were similar in demographic profile, although Group 1 were more likely to be female (odds ratio [OR] 0.65, 95% confidence interval [CI] 0.46–0.93, *p* = 0.018). Those in Group 2 were more likely to undergo a UKA procedure (2/244 Group 1 vs. 13/269, *p* = 0.021), and were significantly less likely have a history of anxiety or depression (OR 0.40, 95% CI 0.25–0.62, *p* < 0.001). There was also a slightly lower BMI value for Group 2, but this was not thought to be clinically relevant (Group 1 30.5 vs. Group 2 29.0, *p* < 0.001). Full details of the demographic comparisons are shown in Table 1.

**TABLE 1:** Demographic comparisons between the two HRQoL groups

Variable	Group 1 (lower EQ‐5D)	Group 2 (higher EQ5D)	Mean difference (MD; 95% CI)/odds ratio (OR; 95% CI)	*p*‐value
Age, years (SD)^a^	69.3 (9.6)	69.6 (9.4)	−0.31; −1.96 to 1.34	0.713
Sex = female				
OR for Grp 2 inclusion^b^	154 (63.0%)	142 (52.7%)	OR 0.65; 0.46–0.93	0.018
SIMD, median	8	8	OR 1.05; 0.98–1.13	0.186
Operation type, *n*(%)^c^	THA 121 (49.5);	THA 133 (50.5);	Reference	
	TKA 121 (49.6);	TKA 123 (50.4);	OR 0.93; 0.65–1.31	0.663
	UKA 2 (13.3)	UKA 13 (86.7)	OR 5.91; 1.31–26.7	0.021
History of anxiety and depression. OR for Grp 2 inclusion^b^	70 (28.6%)	37 (13.8%)	OR 0.40; 0.25–0.62	<0.001
Employment status, *n*(%)^c^	Unemployed 20 (55.6)	Unemployed 16 (44.4)	Reference	
	Employed 51 (42.3)	Employed 67 (56.8)	OR 1.64; 0.78–3.49	0.196
	Retired 166 (48.7)	Retired 175 (51.3)	OR 1.32; 0.66–2.63	0.434
ASA grade, *n*(%)^c^	I 19 (8.6)	I 25 (10.3)	Reference	
	II 141 (63.5)	II 172 (70.8)	OR 0.93; 0.49–1.75	0.816
	III 60 (27.0)	III 44 (18.1)	OR 0.56; 0.27–1.14	0.108
	IV 2 (0.9)	IV 2 (0.8)	OR 0.76; 0.98–5.90	0.793
BMI, mean(SD)^a^	30.5 (5.5)	29.0 (5.1)	1.52; 0.60–2.45	<0.001

Abbreviation: UKA, Unicompartmental knee arthroplasty.

^a^ Independent *t* test.

^b^ Logistic regression.

^c^ Chi‐squared with Bonferroni correction.

^d^ UKA Significantly different to both THA and TKA for Group 2 versus Group 1.

### 3.2 Generalised HRQoL

Regarding the primary outcome of EQ‐5D‐3L MCID attainment there was a significantly lower likelihood of achieving the MCID for those in Group 2 compared to Group 1 (OR 0.13, 95% CI 0.07–0.23, *p* < 0.001). 93.4% of patients achieved the MCID for Group 1, compared to 65.0% for Group 2. The findings remained significant when adjusted for age, gender, history of anxiety/depression, work status, ASA grade, SIMD and pre‐operative OHS/OKS scores (OR 0.07, 95% CI 0.03–0.17, *p* < 0.001). In addition, those in Group 2 had a significantly lower overall improvement in EQ‐5D‐3L scores post‐operatively (Median Group 1 0.67 vs. Median Group 2 0.19; *p* < 0.001).

### 3.3 Joint specific HRQoL

Those in Group 1 had significantly lower pre‐operative OHS/OKS scores than Group 2 (14.1 vs. 23.9 respectively; *p* < 0.001). Post‐operatively there was a significantly smaller increase in OHS/OKS for those in Group 2 compared to Group 1 (Mean 16.4 vs. 22.4 respectively, *p* < 0.001). There was a trend towards lower OKS/OHS MCID attainment for Group 2 compared to Group 1, but this did not reach statistical significance (OR 0.66, 95% CI 0.37–1.19, *p* = 0.168). There was however high rates of OHS/OKS MCID attainment within both groups (91.8% and 88% for Group 1 and Group 2, respectively). The mean difference (MD) between the two groups in final OHS/OKS scores was 3.8 in favour of Group 2 (MD 3.81, 95% CI 2.23–5.43, *p* < 0.001).

### 3.4 Length of stay

Those in Group 2 had a statistically significant shorter length of inpatient hospital post‐operatively (Group 1 mean rank 238 vs. Group 2 mean rank 276; *p* = 0.003). The median length of stay for both groups however was three nights, indicating this difference was likely attributable for more longer length of stay outliers in Group 1. This finding was consistent when a logistic regression model was applied comparing the two groups across the median length of stay. This identified that those in Group 1 were 1.6 times more likely to have a length of stay longer than the median compared to Group 2 (OR 1.65, 95% CI 1.16–2.35, *p* < 0.005).

### 3.5 Clinical outcomes

There were no significant differences between the two groups in readmission at either 30 days (OR Group 2 1.01, 95% CI 0.40–2.52, *p* = 0.986) or 1 year post‐operatively (OR Group 2 0.79, 95% CI 0.45–1.40, *p* = 0.418). There were also no significant differences in reoperation between the two groups at either 30 days (OR Group 2 0.60, 95% CI 0.10–3.63, *p* = 0.580) or 1 year (OR Group 2 1.53, 95% CI 0.55–4.27, *p* = 0.416) post‐operatively.

## 4 DISCUSSION

Overall, we found that patients in Group 1 (those awaiting hip and knee arthroplasty with a lower pre‐operative HRQoL) had greater gains in their generic and joint specific HRQoL following surgery compared to those with higher pre‐operative scores, and that they were around eight times more likely to obtain the MCID post‐operatively. Furthermore, minimal important demographic differences existed between the two groups that may otherwise impact decision making regarding surgical priority (e.g., age or employment status). This raises the possibility that a simple cut off of an EQ‐5D‐3L score of ≤0.487 could help assist decision making for hip and knee arthroplasty prioritisation in the current setting of mismatch between demand and capacity. It is however important to recognise that both groups were likely to have significant improvement in joint specific and generic HRQoL following surgery. There was a significantly higher overall joint specific score found within Group 2 post‐operatively, but this did not meet the MCID.

Our findings support that of Gwynne‐Jones et al. (2020), who also identified that those with lower HRQoL had greater improvements post‐operatively. They however focussed on joint specific scores (OKS/OHS), which may be less informative about the impact of a patient's condition on their overall health and quality of life, compared to EQ‐5D‐3L. This is why more generalised measures (such as EQ‐5D‐3L) have been suggested to provide a better overall reflection of health recovery, for example, after hip fracture (Parsons et al., 2014). Use of EQ‐5D‐3L scores also has the additional benefit of linkage to health economic analysis, where it had been previously demonstrated that procedures in those with lower HRQoL were more likely to be cost‐effective and provide greater cost per quality adjusted life year (QALY; Jenkins et al., 2013).

One of the common complaints regarding the use of generalised (non‐joint specific) HRQoL scores is that the constituent domains may not accurately reflect impact from a single joint pathology. Whilst this is partially true (e.g., in the setting of bilateral hip arthritis), our findings demonstrate a clear and appreciable greater improvement in EQ‐5D‐3L for those with worse pre‐operative scores. This was true even when adjusted for pre‐operative OHS/OKS scores, indicating this improvement is independent of joint disease severity and likely reflects significant variability in how individual patients experience the impact of these symptoms on their overall quality of life.

Strengths of our study include use of widely available and applicable general and joint specific HRQoL scores to determine the potential benefit of using the EQ‐5D‐3L generated dichotomisation of patients for arthroplasty prioritisation, including adjustment for clinically relevant confounders. Due to limitations in the data, we were unable to analyse the potential impact on EQ‐5D‐5L MCID attainment, and this merits consideration and further research to evaluate its use in this setting. Previous work has found that despite significant potential combinations of the individual EQ‐5D‐3L component scores the majority of these are not utilised (Parkin et al., 2010). It is therefore likely that small changes in the score may significantly alter the patient grouping. However, any small change in score is attributable to a large change in symptom mapping—for example, level 1 for pain is classified as ‘extreme’, compared to ‘moderate’ for level 2. Further investigation of the EQ‐5D‐5L may be of interest as this would potentially provide more stable groupings if any domain was to change by a single level. Similar clustering to that used in our study for the dichotomisation has already been demonstrated within the EQ‐5D‐5L (Feng et al., 2016).

Other factors may also play a role in determining patient priority. Prolonged waiting times for example, have been shown to have a significant negative health and economic impact on patients (Nikolova et al., 2016; Ostendorf et al., 2004). Those of a younger age are also more likely to see greater benefit from their health improvement associated with joint arthroplasty (Jenkins et al., 2013; Lalani et al., 2019; Lee et al., 2020). Individuals with problems where delay is likely to lead to significant deterioration and increase surgical complexity, for example, those with rapid radiological change (Reijman et al., 2005; Rosenberg et al., 1992) or inflammatory arthritis (Chmell & Scott, 1999) may also require prioritisation. Finally, a recent study by Al‐Hourani et al. (2021) has also suggested the importance of highlighting those patients currently employed with intent to return to work after surgery. Those in carer roles may also need similar priority given the societal benefits they would offer. Further studies are urgently required to determine which of these factors and other factors should influence surgical prioritisation, as identified by key stakeholders. It is also important to also recognise that general and joint specific HRQoL is likely to change over time and therefore dynamic assessment whilst patients are on the waiting list is required to ensure equality in prioritisation is maintained.

## 5 CONCLUSION

In the unenviable state of necessary healthcare rationing, use of generalised HRQoL measures may help to assist with patient prioritisation. This is evidenced by the fact that those with a lower HRQoL (EQ‐5D‐3L TTO ≤ 0.487) were likely to have greater generalised and joint specific improvements following surgery and were independently more likely to achieve the MCID for EQ‐5D TTO. Further work is required to evaluate if these findings are consistent when using EQ‐5D‐5L and also determine which other factors should be utilised to determine patient priority, including how these factors should be weighed against each other.

## AUTHOR CONTRIBUTION

L. Farrow – Conceptualisation; Formal analysis; Investigation; Methodology; Writing – original draft; Writing – review & editing. J. Redmore – Data curation; Investigation; Writing – original draft; Writing – review & editing. P. Talukdar – Data curation; Formal analysis; Methodology; Resource provision; Writing – review & editing. N. Clement – Conceptualisation; Methodology; Supervision; Writing – review & editing. G. P. Ashcroft – Conceptualisation; Methodology; Supervision; Writing – review & editing

## CONFLICTS OF INTEREST

James Redmore, Partha Talukdar, Nick Clement and George Patrick Ashcroft declare that they have no conflict of interest.

## ETHICS STATEMENT

The data are not publicly available due to privacy or ethical restrictions.

## Data Availability

The metadata that support the findings of this study are available on request from the corresponding author.

## References

[msc1645-bib-0001] Al‐Hourani, K. , MacDonald, D. J. , Turnbull, G. S. , Breusch, S. J. , & Scott, C. E. H. (2021). Return to work following total knee and hip arthroplasty: The effect of patient intent and preoperative work status. The Journal of Arthroplasty *,* 36(2), 434–441. 10.1016/j.arth.2020.08.012 32873451

[msc1645-bib-0002] Carr, A. , Smith, J. A. , Camaradou, J. , & Prieto‐Alhambra, D. (2021). Growing backlog of planned surgery due to covid‐19. BMJ *,* 372, n339. 10.1136/bmj.n339 33563590

[msc1645-bib-0003] Chmell, M. J. , & Scott, R. D. (1999). Total knee arthroplasty in patients with rheumatoid arthritis. An overview. Clinical Orthopaedics and Related Research *,* (366), 54–60. 10.1097/00003086-199909000-00008 10627718

[msc1645-bib-0004] Clement, N. D. , MacDonald, D. , & Simpson, A. H. (2014). The minimal clinically important difference in the oxford knee score and short form 12 score after total knee arthroplasty. Knee Surgery, Sports Traumatology, Arthroscopy: Official Journal of the ESSKA *,* 22(8), 1933–1939. 10.1007/s00167-013-2776-5 24253376

[msc1645-bib-0005] Clement, N. D. , Scott, C. E. H. , Murray, J. R. D. , Howie, C. R. , & Deehan, D. J. , & IMPACT‐Restart Collaboration . (2021). The number of patients “worse than death” while waiting for a hip or knee arthroplasty has nearly doubled during the COVID‐19 pandemic. The Bone & Joint Journal *,* 103‐B(4), 672–680. 10.1302/0301-620X.103B.BJJ-2021-0104.R1 33752468

[msc1645-bib-0006] COVIDSurg Collaborative . (2020). Elective surgery cancellations due to the COVID‐19 pandemic: Global predictive modelling to inform surgical recovery plans. British Journal of Surgery *,* 107(11), 1440–1449. 10.1002/bjs.11746 32395848 PMC7272903

[msc1645-bib-0007] Dolan, P. (1997). Modeling valuations for EuroQol health states. Medical Care *,* 35(11), 1095–1108. 10.1097/00005650-199711000-00002 9366889

[msc1645-bib-0008] EuroQol Group . (1990). EuroQol‐‐a new facility for the measurement of health‐related quality of life. Health Policy, 16(3), 199–208. 0168‐8510. 10.1016/0168-8510(90)90421-9 10109801

[msc1645-bib-0009] Farrow, L. , Gardner, W. T. , Tang, C. C. , Low, R. , Forget, P. , & Ashcroft, G. P. (2021). Impact of COVID‐19 on opioid use in those awaiting hip and knee arthroplasty: A retrospective cohort study. *BMJ Quality & Safety* , bmjqs‐2021‐013450. 10.1136/bmjqs-2021-013450 34521769

[msc1645-bib-0010] Feng, Y. , Devlin, N. , Bateman, A. , Zamora, B. , & Parkin, D. (2016). Prm75 ‐ the distribution of the eq‐5d‐5l index in patients populations. Value in Health *,* 19(3), A84. 10.1016/j.jval.2016.03.1787 30832974

[msc1645-bib-0011] Gutacker, N. , Siciliani, L. , & Cookson, R. (2016). Waiting time prioritisation: Evidence from England. Social Science & Medicine *(1982),* 159, 140–151. 10.1016/j.socscimed.2016.05.007 27183130

[msc1645-bib-0012] Gwynne‐Jones, D. P. , Sullivan, T. , Wilson, R. , & Abbott, J. H. (2020). The relationship between preoperative oxford hip and knee score and change in health‐related quality of life after total hip and total knee arthroplasty: Can it help inform rationing decisions? Arthroplasty Today *,* 6(3), 585–589.e1. 10.1016/j.artd.2020.04.009 32995405 PMC7502579

[msc1645-bib-0013] Jenkins, P. J. , Clement, N. D. , Hamilton, D. F. , Gaston, P. , Patton, J. T. , & Howie, C. R. (2013). Predicting the cost‐effectiveness of total hip and knee replacement: A health economic analysis. The Bone & Joint Journal *,* 95‐B(1), 115–121. 10.1302/0301-620X.95B1.29835 23307684

[msc1645-bib-0014] Lalani, A. , Lee, Y. Y. , Pitta, M. , Westrich, G. H. , & Lyman, S. (2019). Age‐related decline in patient‐reported outcomes 2 and 5 years following total hip arthroplasty. The Journal of Arthroplasty *,* 34(9), 1999–2005. 10.1016/j.arth.2019.02.023 30979671

[msc1645-bib-0015] Learmonth, I. D. , Young, C. , & Rorabeck, C. (2007). The operation of the century: Total hip replacement. Lancet (London, England) *,* 370(9597), 1508–1519. 10.1016/S0140-6736(07)60457-7 17964352

[msc1645-bib-0016] Lee, S. H. , Kim, D. H. , & Lee, Y. S. (2020). Is there an optimal age for total knee arthroplasty?: A systematic review. Knee Surgery & Related Research *,* 32(1), 60–61. 10.1186/s43019-020-00080-1 33198817 PMC7667791

[msc1645-bib-0017] Luo, N. , Johnson, J. , & Coons, S. J. (2010). Using instrument‐defined health state transitions to estimate minimally important differences for four preference‐based health‐related quality of life instruments. Medical Care *,* 48(4), 365–371. 10.1097/mlr.0b013e3181c162a2 20355266

[msc1645-bib-0018] Murray, D. W. , Fitzpatrick, R. , Rogers, K. , Pandit, H. , Beard, D. J. , Carr, A. J. , & Dawson, J. (2007). The use of the oxford hip and knee scores. The Journal of Bone and Joint Surgery. British Volume *,* 89(8), 1010–1014. 10.1302/0301-620X.89B8.19424 17785736

[msc1645-bib-0019] NHS England (2021). *2021/22 p* riorities and operational planning guidance: Implementation guidance. NHS. https://www.england.nhs.uk/wp‐content/uploads/2021/03/B0468‐implementation‐guidance‐21‐22‐priorities‐and‐operational‐planning‐guidance.pdf

[msc1645-bib-0020] Nikolova, S. , Harrison, M. , & Sutton, M. (2016). The impact of waiting time on health gains from surgery: Evidence from a national patient‐reported outcome dataset. Health Economics *,* 25(8), 955–968. 10.1002/hec.3195 26013773

[msc1645-bib-0021] Ostendorf, M. , Buskens, E. , v an Stel, H. , Schrijvers, A. , Marting, L. , Dhert, W. , & Verbout, A. (2004). Waiting for total hip arthroplasty: Avoidable loss in quality time and preventable deterioration. The Journal of Arthroplasty *,* 19(3), 302–309. 10.1016/j.arth.2003.09.015 15067641

[msc1645-bib-0022] Oussedik, S. , MacIntyre, S. , Gray, J. , McMeekin, P. , Clement, N. D. , & Deehan, D. J. (2021). Elective orthopaedic cancellations due to the COVID‐19 pandemic: Where are we now, and where are we heading? Bone & Joint Open *,* 2(2), 103–110. 10.1302/2633-1462.22.BJO-2020-0161.R1 33573397 PMC7925214

[msc1645-bib-0023] Parkin, D. , Rice, N. , & Devlin, N. (2010). Statistical analysis of EQ‐5D profiles: Does the use of value sets bias inference? Medical Decision Making *,* 30(5), 556–565. 10.1177/0272989X09357473 20228285

[msc1645-bib-0024] Parsons, N. , Griffin, X. L. , Achten, J. , & Costa, M. L. (2014). Outcome assessment after hip fracture. Bone & Joint Research *,* 3(3), 69–75. 10.1302/2046-3758.33.2000250 24648420 PMC3963508

[msc1645-bib-0025] Reijman, M. , Hazes, J. M. , Pols, H. A. , Bernsen, R. M. , Koes, B. W. , & Bierma‐Zeinstra, S. M. (2005). Role of radiography in predicting progression of osteoarthritis of the hip: Prospective cohort study. BMJ *,* 330(7501), 1183. 10.1136/bmj.38442.457488.8F 15894555 PMC558014

[msc1645-bib-0026] Rosenberg, Z. S. , Shankman, S. , Steiner, G. C. , Kastenbaum, D. K. , Norman, A. , & Lazansky, M. G. (1992). Rapid destructive osteoarthritis: Clinical, radiographic, and pathologic features. Radiology *,* 182(1), 213–216. 10.1148/radiology.182.1.1727284 1727284

[msc1645-bib-0027] Yapp, L. Z. , Clarke, J. V. , Moran, M. , Simpson, A. H. R. W. , & Scott, C. E. H. (2021). National operating volume for primary hip and knee arthroplasty in the COVID‐19 era: A study utilizing the Scottish arthroplasty project dataset. Bone & Joint Open *,* 2(3), 203–210. 10.1302/2633-1462.23.BJO-2020-0193.R1 33739125 PMC8009902

[msc1645-bib-0028] Yeo, M. G. H. , Goh, G. S. , Chen, J. Y. , Lo, N. N. , Yeo, S. J. , & Liow, M. H. L. (2020). Are Oxford hip score and Western Ontario and McMaster universities osteoarthritis index useful predictors of clinical meaningful improvement and satisfaction after total hip arthroplasty? The Journal of Arthroplasty *,* 35(9), 2458–2464. 10.1016/j.arth.2020.04.034 32416955

